# Fine mapping and identification of candidate genes associated with powdery mildew resistance in melon (*Cucumis melo* L.)

**DOI:** 10.1093/hr/uhae222

**Published:** 2024-08-12

**Authors:** Xiaoyu Duan, Yue Yuan, Núria Real, Mi Tang, Jian Ren, Jiaqi Wei, Bin Liu, Xuejun Zhang

**Affiliations:** Hami-melon Research Center, Xinjiang Academy of Agricultural Sciences, Nanchang Road 403, Saybagh District, Urumqi 830091, China; Sanya Mingzhu Melon and Watermelon Variety Demonstration Evaluation and Research Center, Yazhou District, Sanya 572000, China; Wuhan Academy of Agricultural Sciences, Baishazhou Avenue 107, Hongshan District, Wuhan 430072, China; Plant Pathology, IRTA Cabrils. Carretera de Cabrils km 2, 08348 Cabrils Spain; Wuhan Academy of Agricultural Sciences, Baishazhou Avenue 107, Hongshan District, Wuhan 430072, China; Anhui Jianghuai Horticulture Seeds Co., Ltd, Changfeng County, Hefei City, Anhui Province; Wuhan Academy of Agricultural Sciences, Baishazhou Avenue 107, Hongshan District, Wuhan 430072, China; Wuhan Academy of Agricultural Sciences, Baishazhou Avenue 107, Hongshan District, Wuhan 430072, China; Hami-melon Research Center, Xinjiang Academy of Agricultural Sciences, Nanchang Road 403, Saybagh District, Urumqi 830091, China; Sanya Mingzhu Melon and Watermelon Variety Demonstration Evaluation and Research Center, Yazhou District, Sanya 572000, China; Hami-melon Research Center, Xinjiang Academy of Agricultural Sciences, Nanchang Road 403, Saybagh District, Urumqi 830091, China; Sanya Mingzhu Melon and Watermelon Variety Demonstration Evaluation and Research Center, Yazhou District, Sanya 572000, China; Hainan Sanya Crops Breeding Trial Center of Xinjiang Academy Agricultural Sciences, Haitang District, Sanya, 572000

## Abstract

Powdery mildew (PM), a common disease of many major crop species, including melon (*Cucumis melo* L.), affects plant growth and fruit quality and seriously reduces production. Using a combined morphological and molecular approach, we attribute the PM pathogen that naturally occurs in melon to *Podosphaera xanthii*, and specifically to physiological race 1. An investigation into the genetic basis of PM resistance in melon using the resistant accession ‘PI 164637’ and susceptible counterpart ‘HDZ’ reveals dominant inheritance of PM resistance at the seedling stage, supported by F_2_ and backcross population segregation ratios. Adult plant assessments indicate a major gene with an additive effect for PM resistance. Bulk segregant analysis coupled with high-throughput sequencing identified a significant quantitative trait locus on chromosome 6 that is associated with PM resistance. Genetic mapping narrowed down the candidate region to 63.5 kb using InDel molecular markers, harboring 12 candidate genes. The marker chr06_indel_5 047 127 demonstrated high accuracy in screening PM resistance in an F_2_ segregating population and 30 inbred lines as natural populations. Functional annotation and expression analysis of candidate genes revealed that MYB transcription factor MELO3C006700, GATA transcription factor MELO3C028829 and heparanase-like protein MELO3C006697 are promising candidate genes for PM resistance in melon. The genetic architecture underlying this resistance in melon offers valuable insights for breeding programs, and the identified markers, especially chr06_indel_5 047 127, may enable practical applications for marker-assisted selection in developing PM-resistant melon varieties.

## Introduction

Melon (*Cucumis melo* L., Cucurbitaceae; 2n = 2x = 24) is an economically valuable, widely cultivated horticultural species with diverse commercially attractive properties that are popular among consumers (e.g. flesh color, texture, flavor, high nutritional content) [1, 2]. Melon is usually cultivated in fields and greenhouses in temperate and tropical regions. However, these regions often experience a proliferation of powdery mildew (PM) due to the relative high humidity and temperature.

PM occurs worldwide and poses a threat to yield and quality of crop species, vegetables, and fruits such as rice, wheat, pepper, grape, and melon [3–6]. PM within the Cucurbitaceae is primarily caused by the biotrophic fungi *Podosphaera xanthii* and *Golovinomyces cichoracearum* [7]. These two species that target leaves, stems, and even the fruit, which often develop white spots because of hyphal growth, disrupting photosynthesis, and reducing fruit yield and quality [8–10]. Consequently, interest in identifying sources of PM resistance is increasing.

*Podosphaera xanthii* tends to prevail in tropical and subtropical areas, whereas *G. cichoracearum* predominates in temperate regions [11]. The first record of *P. xanthii* dates to 1925 in California, but numerous physiological races have been identified since (e.g. 0, 1, 2F, 2US, 3, 4, 5, N1 (race 6), N2 (race 7), N3, N4) [12]. These variants are influenced by differences in climate, geography, and melon cultivar and lead to distinct manifestations of PM in different countries. For example, races 1–3 and S have been identified in the USA [13, 14]; race 1, 2, and 4 are prevalent in Brazil [15–17]; N1, N2, N3, and N4 have been identified in Japan [18]; KN1 and KN2 have been identified in South Korea; and 1 and 2F have been reported from China [19–21]. In contrast, *G. cichoracearum* predominantly causes PM in the Czech Republic [22].

Bulked segregant analysis (BSA) is an important approach in molecular marker-associated breeding. It is used to pinpoint target genes or genomic regions by two bulks with highly segregating populations [23]. Rapid advancements in Next-Generation Sequencing (NGS) have enabled BSA to be combined with NGS. This integration allows for whole-genome sequencing and single-nucleotide polymorphism (SNP) analysis, significantly accelerating identification of target genes or genomic regions [24].

NGS-based BSA methods have identified numerous genes and quantitative trait locus (QTLs) responsible for conferring resistance to PM in melon. For instance, the *Pm-8* locus identified in ‘PI 134198’ accession confers resistance to a unique *P. xanthii* race, *PxCh1*, which is prevalent around Shanghai [25]. Another example includes the *Pm-2F* locus, which has been implicated in resistance to *P. xanthii* race 2F in the ‘K7–1’ accession [20]. Additionally, a major QTL named *Bpm12.1* was detected, encompassing 17 genes, seven of which were potentially involved in PM resistance in the ‘MR-1’ accession [26]. Two PM-resistant loci, *Pm2.1* and *Pm12.1,* were then identified and mapped to chromosomes 2 and 12, respectively, in the ‘PMR6’ accession [27]. Recently, a novel QTL (*qCmPMR-12*) associated with PM resistance was identified and mapped to chromosome 12, where *MELO3C002434* was identified as a putative candidate gene related to PM resistance through further RNA-seq analysis [28].

We investigate the genetic basis of PM resistance in the ‘PI 164637’ melon accession, identify candidate genes, and develop molecular markers for subsequent application in breeding melon varieties with PM resistance. We construct genetic populations and use BSA-seq and NGS to identify a major QTL, map candidate genes responsible for PM resistance in melon, and develop molecular markers related to PM resistance. These markers could be applied to select melon varieties with PM resistance.

## Results

### *Podosphaera xanthii* causes PM in susceptible melon accession ‘HDZ’

**Figure 1:**
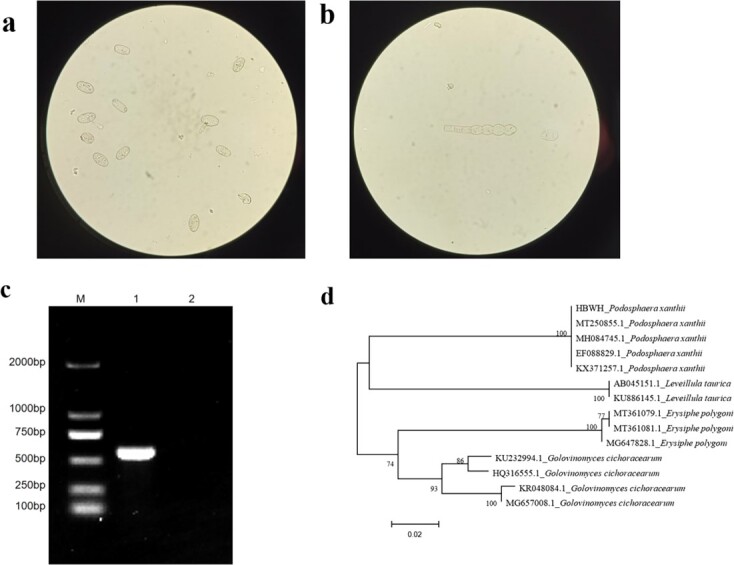
Morphological and molecular identification of PM in melon. (a) Morphological observation of conidia and (b) conidiophores under 10 × 40 field of view. (c) DNA amplification of PM, where M represents the molecular weight marker; ‘1’ represents the PCR product of PM; ‘2’ represents the negative control. (d) Phylogenetic analysis of the ITS sequence of *P. xanthii*, *G. cichoracearum, E. polygoni*, *L. taurica*.

The morphology of the PM pathogen was examined microscopically, revealing oval conidia with fibrous bodies and side germination (Fig. 1a). The conidial stalk has a moniliform appearance (Fig. 1b). These morphological characteristics are consistent with *P. xanthii*. Pathogen DNA was extracted from completely infected leaves and amplified using specific primers. A sequence of ~540 bp (designated HBWH-*P. xanthii*) was produced (Fig. 1c), which, when submitted to the NCBI database for BLAST analysis, was 100% identical to *P. xanthii* from various species such as pumpkin (MT250855.1), squash (MH084745.1), and okra (MH824669.1). Phylogenetic tree construction confirmed its clustering with other *P. xanthii* sequences (Fig. 1d).

### Physiological race 1 of *P. xanthii* causes PM infection in melon

**Figure 2:**
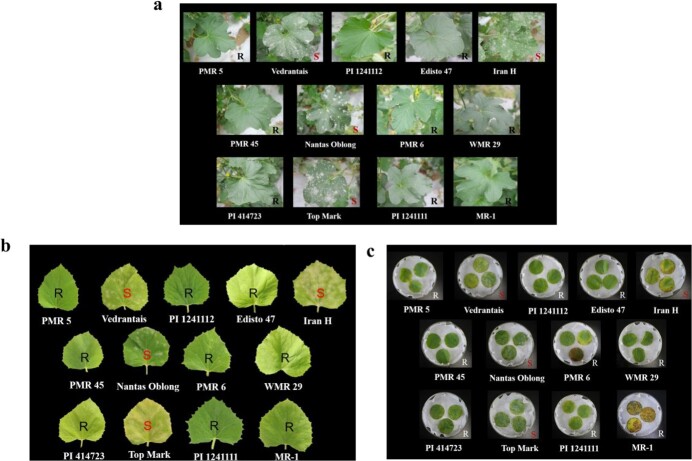
Reactions of 13 host melon accessions to PM. Reponses in either living plants (a), living leaves (b), or detached leaves (c).

To accurately determine the physiological race of *P. xanthii*, reactions of 13 host accessions to PM were assessed using three methods, and the disease index (*DI)* was calculated. Although slight variation in the *DI* occurred for the same accession inoculated by different methods (Table S1), resistance determination remained consistent across them. Specifically, ‘Iran H’, ‘Top Mark’, ‘Védrantais’, and ‘Nantais Oblong’ were susceptible, while other accessions exhibited resistance (Fig. 2a–c). Based on previous findings, the accession PMR 45 was resistant to race 1, but susceptible to race 2 [13]. Therefore, we identify the physiological race of *P. xanthii* in our study to be race 1.

### PM resistance in melon ‘PI 164637’presents a dominant inheritance

Resistance evaluation was performed at both seedling and adult stages on genetic populations using the *DI* to determine disease severity. At the seedling stage, female ‘PI 164637’ and male ‘HDZ’ parents were identified as highly resistant (HR) and sensitive (S), respectively, while the F_1_ was identified as HR (Table 1), suggesting that PM resistance from ‘PI 164637’ was governed by dominant genes. Analysis of the F_2_ segregating population revealed an expected ratio of 3:1 for resistance to susceptible plants (χ^2^ = 0.07). In the BCP_1_ population, all plants exhibited resistance, while in the BCP_2_ population, with an expected ratio of 1:1 for resistant to susceptible plants (χ^2^ = 0.04) (Table 2), these findings suggest that the PM resistance from ‘PI 164637’ was governed by a single dominant gene.

**Table 1:** Genetic analysis of PM resistance in the genetic populations during the seedling stage

Generation	Resistance			Susceptibility		Expected susceptible: resistant ratio	χ^2^ value	*P-*value
	IM	HR	R	S	HS			
PI 164637 (P_1_)	20	0	0	0	0			
HDZ (P_2_)	0	0	0	20	0			
F_1_	0	10	0	0	0			
F_2_	36	41	18	23	5	3:1	0.07	0.79
BCP_1_	19	23	1	0	0			
BCP_2_	0	7	20	11	12	1:1	0.04	0.84

**Table 2:** Validation of InDel molecular markers in the F_2_ population

Marker	Band type	Number of F_2_ plants	Number of resistant plants	Accuracy (%)
chr06_indel_5 047 127	A	108	108	100
	B	106	55	48.11
chr06_indel_5 110 650	A	110	109	99.10
	B	106	56	47.17

A: PM-resistant type; B: PM-susceptible type.

**Figure 3:**
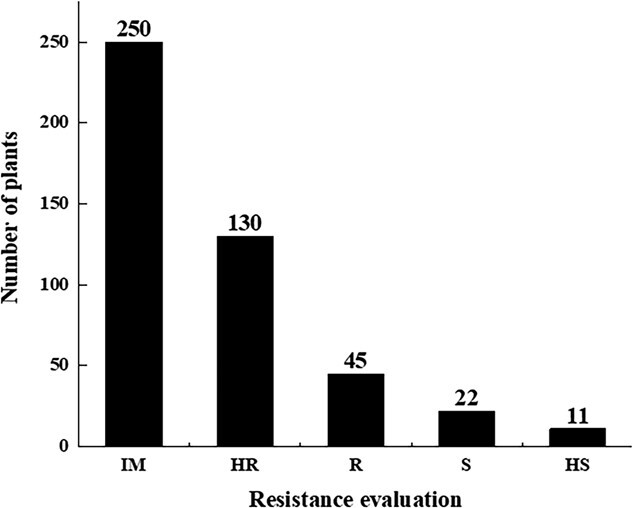
Evaluation of PM resistance in the F_2_ segregating population of ‘PI 16437’ × ‘HDZ’. IM: immune to PM; HR: high resistance to PM; H: moderate resistance to PM; S: susceptibility to PM; HS: high susceptibility to PM.

During evaluation of PM resistance in adult plants, the leaves of the female parent ‘PI 164637’ presented no white spots, which are classified as immune (IM). The *DI* of the male parent ‘HDZ’ was 76.3 and was classified as S; the *DI* of the F_1_ population was 4, indicating that it was HR. In the F_2_ segregating population, the ratio of resistant to susceptible plants was 425:33 (Fig. 3). A major gene plus polygene genetic model was then used in the F_2_ segregating population to elucidate the genetic inheritance of PM resistance. Next, a genetic model was determined using Akaike Information Criterion (AIC) values. Based on the lowest AIC values, the ‘one major gene with an additive effect’ (1MG-A) model was selected as the most suitable for PM resistance (Tables S2 and S3). This result implies that resistance from ‘PI 164637’ accession adheres to an additive effect model controlled by one major gene.

### BSA-seq analysis identified a significant QTL on chromosome 6 associated with PM resistance in melon

**Figure 4:**
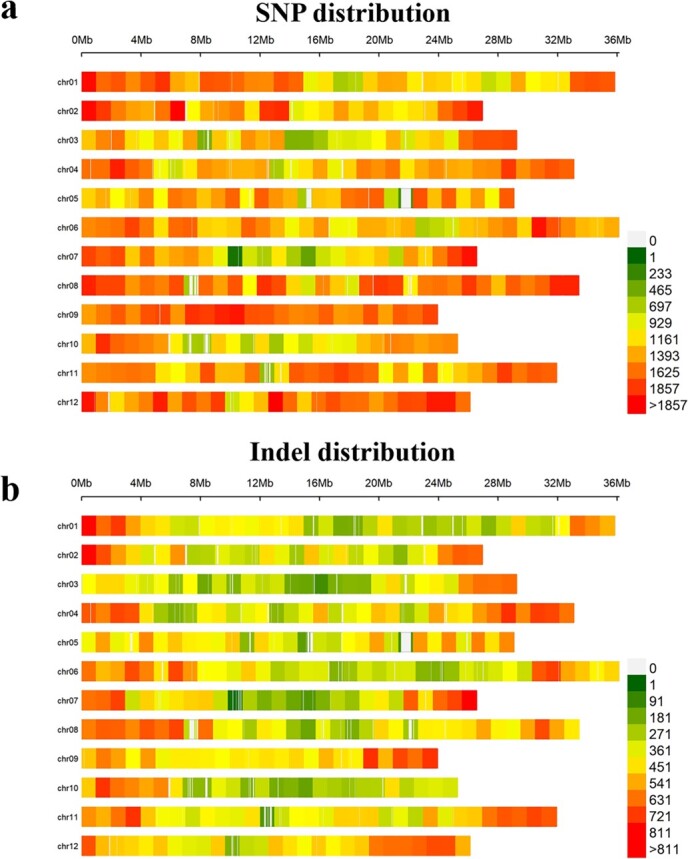
Distribution of SNPs (a) and Indels (b) across the 12 chromosomes in melon. Colors and numbers represent the density of SNPs and InDels on each chromosome.

To further characterize PM resistance and identify candidate genomic regions or genes associated with it, we conducted a BSA-seq analysis using parental lines and two composite pools of 25 F_2_ individuals. A total of 61.85 Gb of clean reads were acquired, with Q20 and Q30 values >96.77% and 91.31%, respectively. The GC average content was 37%, confirming the high-quality nature of sequence data for subsequent analysis (Table S4). Subsequently, 511 387 SNPs and 157 785 InDels were identified between ‘PI 164637’ and ‘HDZ’ accessions. These SNPs were evenly distributed across each chromosome, indicating their potential value for mapping PM-resistant genes (Fig. 4a, b).

**Figure 5:**
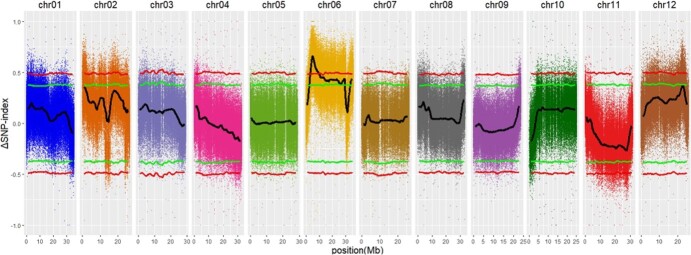
Identification of QTLs for PM resistance in melon. Δ(SNP-index) plot of the 12 chromosomes in the F_2_ segregating population. The red line represents the 99% confidence interval, the green line represents the 95% confidence interval, and the black line represents the ∆(SNP-index). Dots of different colors represent SNPs on different chromosomes.

To assess the disparity in allele frequencies between resistant and susceptible accessions, the Δ(SNP-index) was calculated from the resistant and susceptible bulked pools, with a 99% confidence interval used as a threshold. A single QTL identified on chromosome 6, spanning 3.30–9.80 Mb, was associated with PM resistance (Fig. 5).

### Development of InDel molecular markers for fine mapping of PM resistance

**Figure 6:**
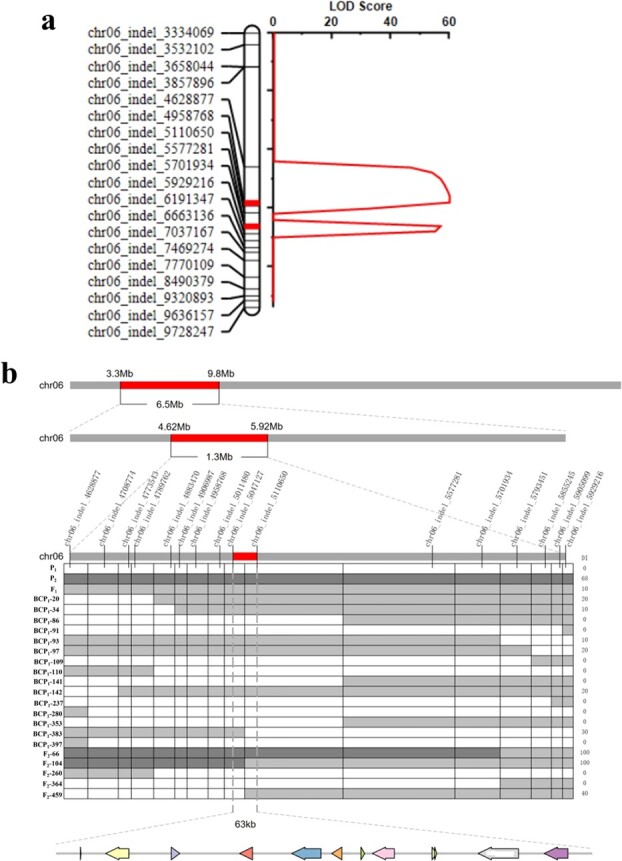
QTL analysis and fine mapping of PM resistance genes on chromosome 6 in melon. (a) Genetic linkage map and QTL analysis in candidate regions. (b) Development of InDel molecular markers and fine mapping of PM-resistant genes on chromosome 6. Different shapes represent different candidate genes in this region.

Based on BSA-seq analysis, a total of 20 InDel molecular markers were developed within the identified candidate region (3.30–9.80 Mb on chromosome 6). These markers were used for polymorphism verification in both parental lines and the F_2_ segregating population. Of these markers, 19 with clear and usable bands were used for genotyping. Genetic mapping construction and QTL analysis within the candidate region were performed using QTL IciMapping software. Consequently, two linkage QTLs were mapped to the region between chr06_Indel_4 628 877 and chr06_Indel_5929216 (from 4 628 877 to 592 916 on chromosome 6), with logarithm of the odds (LOD) scores of 60.67 and 56.63, respectively. The physical distance covered 1.3 Mb and included 32 candidate genes (Fig. 6a). New InDel molecular markers were also developed for further screening in a total of 1040 F_2_ and BCP_2_ segregating populations to narrow down the candidate region of PM resistance. Twenty recombinants were identified during screening. The final candidate region was determined to be between chr06_indel_5 047 127 and chr06_indel_5 110 650 markers (Fig. 6b), spanning 63.5 kb (from 5 047 127 to 5 110 650) on chromosome 6. This region contains 12 candidate genes.

### The molecular marker chr06_indel_5 047 127 for PM resistance demonstrates high accuracy and consistency

To assess the reliability of the developed InDel molecular markers for chr06_indel_5 047 127 and chr06_indel_5 110 650, a validation study was performed using 458 F_2_ segregating individuals. Genotyping accuracy was calculated, revealing a 100% accuracy for chr06_indel_5 047 127 and 99.10% for chr06_indel_5 110 650 (Table 2). The two InDel molecular markers were further validated using 30 inbred lines as natural populations. This set included 12 HR, 5 resistant (R), 9 susceptible (S), and 4 highly susceptible (HS) inbred lines. This analysis identified 9 inbred lines, comprising 3 each of HR and R inbred lines, 2 S inbred lines, and 1 HS inbred line, resulting in an accuracy of 66.67% for screening PM-resistant inbred lines (Table S5). However, chr06_indel_5 110 650 did not effectively screen for PM resistance among the 30 inbred lines, because the expected genetic bands consistent with ‘PI 164637’ did not appear. Therefore, the chr06_indel_5 047 127 molecular marker is more suitable for breeding PM resistance because of its higher accuracy and consistency.

### Functional annotation highlights candidate genes for PM resistance

**Figure 7:**
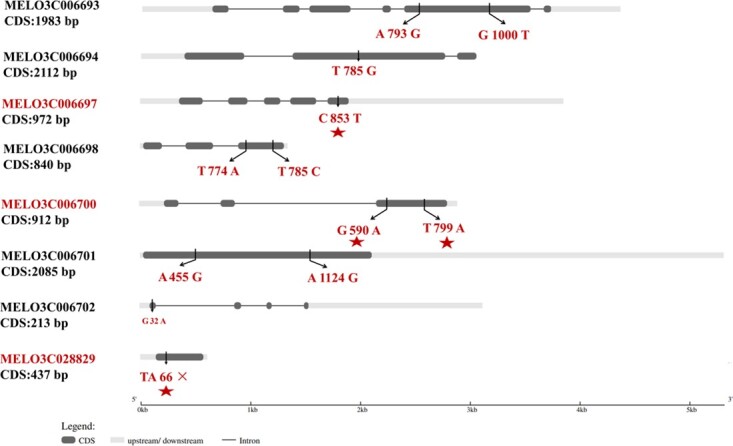
Non-synonymous SNP mutation in candidate genes between two parental lines. The left letter represents base in ‘PI 164637’, the right letter represents base in ‘HDZ’, the number represents location in CDS sequence. Asterisks indicate deleterious SNP substitutions.

To explore the potential involvement of the 12 identified candidate genes, functional annotation and expression analysis were performed. Protein annotations revealed several genes have potential roles associated with disease resistance. Examples included some genes involved in cell wall formation such as *MELO3C006694*, *MELO3C006695*, and *MELO3C006698*, and transcription factors such as *MELO3C006696* and *MELO3C006700* (Table S6). We discarded *MELO3C035461* from subsequent analysis because of its identical annotation and location with *MELO3C028829*. Further, the sequences were analyzed between parents, and the results showed that except for three candidate genes (*MELO3C006695*, *MELO3C006696,* and *MELO3C006699*), other candidate genes all exhibited difference in exon sequence between parents (Fig. 7). It is worth mentioning that *MELO3C028829*, annotated as GATA zinc finger domain-containing protein 10-like isoform X2, presented an early termination, leading to loss of its function in ‘PI 164637’, which could be related to PM resistance in melon (Fig. S1). The amino acid substitutions analysis showed that deleterious substitutions were found in *MELO3C006697* (*P* < 0.05, L285F) and *MELO3C006700* (*P* < 0.05, G197E, and W267K) according to SIFT tools, implying they could participate in PM resistance in melon (Table S7).

**Figure 8:**
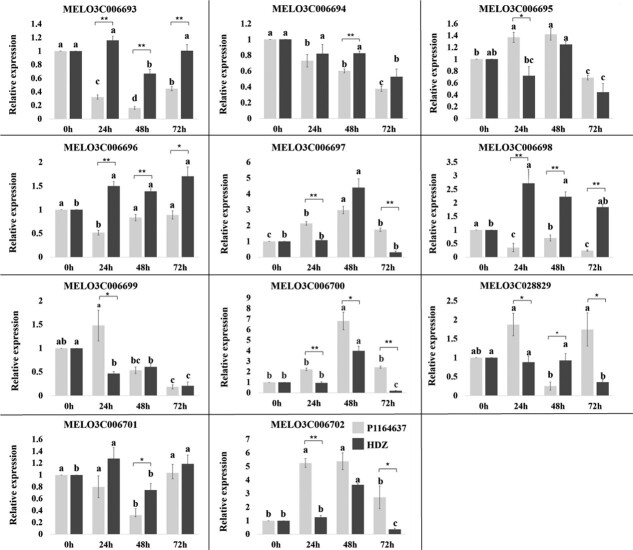
Relative expression of 11 candidate genes at different time points after PM infection in ‘PI 164637’ and ‘HDZ’ accessions (*n* = 3). Asterisks indicate significant differences in gene relative expression between parental accessions (*P* < 0.05).

Additionally, an expression analysis was performed using real-time quantitative PCR (RT-qPCR). Significant differences in expression profiles of these candidate genes were apparent between parents. Seven genes (*MELO3C006695*, *MELO3C006796*, *MELO3C006697*, *MELO3C006699*, *MELO3C006700*, *MELO3C028829*, *MELO3C006702*) exhibited significantly higher relative expression in the resistant parental accession ‘PI 164637’ compared with the susceptible parental ‘HDZ’, while remaining candidate genes showed an opposite trend (Fig. 8). The relative expression of *MELO3C006700* in ‘PI 164637’ have the largest change in relative expression among all the candidate genes suggesting its potential role in PM resistance in melon.

## Discussion

### Importance of confirming *P. xanthii* race 1 as PM causative agent in melon

PM represents a considerable threat to important horticultural crops such as melon, cucumber, and grape [29, 30]. A characteristic symptom of PM is the formation of white spots on leaves, which can spread to other plants and lead to large-scale reductions in growth and fruit quality. The PM pathogen encompasses 18 genera, comprising 873 species, with distinct physiological races identified across different countries or regions [31–33]. Therefore, it is imperative to accurately identify the PM pathogen and its physiological races to effectively manage this disease in melon. Phylogenetic analysis and sequencing play important roles in this process [34]. The recognition of distinct races, particularly race 1 in this study, emphasizes the diversity within the pathogen. Understanding these variations is necessary to devise effective strategies to combat PM, especially considering its potential for rapid spread and substantial crop damage.

### Genetic inheritance in PM resistance in melon ‘PI 164637’

Genetic resistance is a fundamental strategy for controlling PM in various crops, including melon. The complex interplay between genetic and environmental factors presents challenges in elucidating the genetic inheritance of PM, which can vary across species and varieties. For instance, PM resistance in Indian mustard is governed by two semi-dominant genes with gene dosage effects [35]. In cucumber, resistance varies from multiple recessive genes to 1 or 2 major and 1 or 2 minor genes, including scenarios where 1 or 2 genes control resistance [36]. We report PM resistance in ‘PI 164637’ to be governed by a single dominant gene, which is consistent with findings in accessions ‘PMR45’, ‘WMR29’, ‘PI414723’, and ‘MR-1’ [26, 37]. This discovery provides a clear genetic basis for resistance, streamlines breeding efforts, and facilitates development of melon varieties with enhanced resistance through targeted selection and breeding programs.

### QTL analysis in PM resistance in melon ‘PI 164637’

Compared with traditional breeding methods, BSA coupled with NGS has significantly contributed to identification of QTLs and genes associated with PM resistance. Furthermore, the emergence of new physiological races has become an increasingly serious problem, potentially leading to the loss of PM resistance in formerly resistant varieties [38]. Consequently, there is an ongoing need to explore novel PM-resistant loci for breeding melon varieties with sustained PM resistance.

Numerous QTLs and genes related to PM resistance have been identified in specific melon accessions. In the case of melon accession ‘Edisto47’, the candidate genes for PM resistance are *MELO3C015353* and *MELO3C015354*, located on chromosome 2 [39]. In the accession ‘MR-1’, two genes, *CmPMRl* (on chromosome 12) and *CmPMrs* (on chromosome 10), confer PM resistance to leaves and stems, respectively [21]. Our identification of a significant QTL on chromosome 6 enhances our understanding of the genetic architecture underlying PM resistance in melon. This genomic insight enables precise marker-assisted selection, facilitating the development of melon varieties with improved resistance.

### MYB transcription factor MELO3C006700, GATA zinc finger protein MELO3C028829 and heparanase-like protein MELO3C006697: promising candidates for melon defense against PM

We identified 12 candidate genes associated with PM resistance in melon, among which MELO3C028829 presented a shorter protein in PI 164637, and MELO3C006697 and MELO3C006700 presented deleterious substitutions between parents. MELO3C028829 is annotated as GATA zinc finger domain-containing protein 10-like isoform X2. GATA zinc finger domain-containing proteins are transcriptional regulatory proteins functioned in various developmental processes, including chlorophyll biosynthesis and environmental stresses response [40–43]. GATA genes are involved in abiotic and biotic stresses in cucumber [44]. Previous research demonstrated that several cucumber GATA genes, such as *Csa5G622830* and *Csa2G162660*, have altered expression patterns in susceptible and resistant cucumber varieties in response to PM infection [45]. In our study, the truncated protein observed in ‘PI 164637’ suggests a potential advantage by potentially eliminating or modifying a protein domain that positively impacts on the plant defense mechanisms. Thus, MELO3C028829 emerges as a promising candidate. Meanwhile, MELO3C006697, annotated as a heparanase-like protein, possesses deleterious substitutions between parental lines, suggesting a possible alteration in its functionality. Although traditionally associated with extracellular enzymatic roles in signaling pathways regulating cell growth, detoxification, and cell wall fortification [46–49], the specific involvement of heparanase-like proteins in PM resistance necessitates further investigation. Lastly, MELO3C006700, annotated as an MYB transcription factor, exhibits significant upregulation in the resistant parental accession ‘PI 164637’ and harbors deleterious substitutions, which made MELO3C006700 as another very promising candidate in melon defense against PM. MYB transcription factors in other species, such as TuMYB46L-TuACO3 module in wheat and VdMYB1 in grapevine, have been implicated in the regulation of defense mechanisms against PM [50, 51]. These findings underscore the complexity of the genetic mechanisms underlying PM resistance in melon and emphasize the need for further functional studies to elucidate the specific roles of these candidate genes and inform future molecular breeding strategies aimed at enhancing melon resistance to PM.

## Conclusions

We identify *P. xanthii* race 1 as the causative agent of PM in melon, with PM resistance likely controlled by a single dominant gene in the ‘PI 164637’ accession. Using BSA-seq and InDel molecular markers, a 63.2-kb candidate region on chromosome 6 containing 12 candidate genes was identified. Further analysis of relative expression and protein sequences revealed that the MYB transcription factor MELO3C006700, GATA Zinc Finger Protein MELO3C028829, and heparanase-like protein MELO3C006697 are promising candidates for PM resistance in melon. Moreover, the molecular marker ‘chr06_indel_5 047 127’ shows promise for screening PM-resistant melon varieties.

## Materials and methods

### Plant materials

The melon accession ‘PI 164637’ (P_1_), highly resistant to PM, and the accession ‘HDZ’ (P_2_), highly susceptible to PM, were used as female and male parents, respectively (Fig. S2). The accession P_1_ was crossed with P_2_ to produce an F_1_ population, and the resulting F_1_ population was self-crossed to produce an F_2_ segregating population. At the same time, the F_1_ was backcrossed with P_1_ and P_2_ to obtain the BCP_1_ and BCP_2_ populations, respectively. The two parents, along with the F_1_, F_2_, BCP_1_, and BCP_2_ populations, were used to determine and validate the genetic inheritance of PM resistance in melon. Additionally, 13 common melon hosts were used to determine the physiological race of the *P. xanthii* [52]. Thirty inbred lines (CL_1–30) as natural population were used to validate the molecular markers. Seeds were kindly provided by Hami Melon Research Center, Xinjiang Academy of Agricultural Sciences (Urumqi, Xinjiang, China). These plant materials were grown in a plastic greenhouse at Sanya experimental field under conditions of 28°C–30°C/16 h day and 18°C–20°C/8 h night, and clamped with grafting clips after hand pollination. Standard horticultural procedures were implemented for melon plants, including irrigation, fertilization, and pest and disease control.

### Identification of the PM pathogen

Three leaves of ‘HDZ’ naturally infected by PM were collected and immediately taken to the laboratory for PM pathogen purification, as follows: a single white spot was selected and inoculated onto the susceptible accession ‘Iran H’ at the two-true-leaves stage, to enable pathogen propagation. Subsequently, when the plants were fully infected, a new white spot was selected and inoculated onto two fresh leaves on new plants in the two-true-leave stage. This process was repeated three times to ensure purity, and the white spot was used to identify the PM pathogen as well as its physiological race.

For morphological identification of the PM pathogen, a single white spot was selected and resuspended in 1 ml of 0.3% KOH solution. The mixture was then dropped onto a microscope slide to observe pathogen morphology via light microscopy (JNOEC, China). For molecular identification of the PM pathogen, the ITS sequence in the PM pathogen was amplified using a specific primer pair (F: 5’-TCCGTAGGTGAACCTGCGG-3′, R: 5’-TCCTCCGCTTATTGATATGC-3′) and then sequenced. The PCR was conducted with a total volume 50 μl per reaction, comprising an amplification system including 4 μl of DNA template (50 ng·μl^−1^), 2 μl of each primer (10 μM), 25 μl of 2 × Phanta Max Buffer, 1 μl of dNTP Mix, 1 μl of Phanta Max Super-Fidelity DNA Polymerase, and made up to 50 μl using ddH_2_O. The PCR reaction program was performed, and the reaction program involved 94°C for 5 min, followed by 35 cycles of 94°C for 30 s, 55°C for 30 s, 72°C for 45 s, a final amplification of 72°C for 10 min, and storage at 4°C.

### Phylogenetic tree construction

The target ITS sequence, along with other congeneric ITS sequences from the NCBI database, were prepared for phylogenetic tree construction. Other ITS sequences belonging to *P. xanthii*, *G. cichoracearum*, *Erysiphe polygoni*, and *Leveillula taurica* were also downloaded from this database. Multiple sequences were aligned using ClustalW, and the phylogenetic tree was constructed using MAGEX software [53] and a neighbor-joining method with 1000 bootstrap replicates [54, 55].

### Determination of physiological race

The physiological race of the PM pathogen was determined based on the responses of 13 hosts (Iran H, Top Mark, Védrantais, PMR 45, PMR 5, WMR 29, Edisto 47, PI 414723, MR-1, PI 124111, PI 124112, PMR 6, Nantais Oblong) to PM in experiments involving natural occurrence in the field, inoculation to living leaves, and inoculation to detached leaves. When results were consistent across methods, a physiological race was assigned. When results varied, pathogen isolation and purification were performed for characterization. For natural occurrence in the field, 13 host accessions were planted, each consisting of 30 plants. No fungicide was applied throughout the growing stage. PM symptoms were identified upon complete infection, and 10 severely infected leaves from each of nine plants were used to calculate PM incidence. For inoculation of PM to living leaves, the 13 host accessions were planted with each accession, with five plants forming a replicate, with three replicates. When seedings reached the two-true-leaves stage, a spore suspension (1 × 10^5^ spores per ml in 0.05% Tween-20) was sprayed to thoroughly cover the entire plant. After inoculation, plants were placed into a growth chamber and cultured in darkness for 24 h, then into a chamber at 28°C/16 h day and 18°C/8 h night, with 80% relative humidity for 14 days. The first and second true leaves were used to calculate PM incidence. For inoculation of PM to detached leaves, three uninfected leaves from the 13 host accessions were collected and cut into discs of 3-cm diameter. Each replicate had three groups of three discs that were placed into culture dishes with wet filter paper. The inoculation method for detached leaves was identical to that for living leaves, and the investigation of PM incidence was performed 7 days after inoculation.

### Phenotyping

A standardized scale was established to evaluate PM resistance in melons based on the responses of these plants and leaves to the PM pathogen (Table 3).

**Table 3:** Scale for evaluation of PM resistance

Disease grade	Description of symptoms in living plant leaves	Description of symptoms in detached leaves
0	Asymptomatic	Asymptomatic
1	Minor symptoms with <5% of the leaf covered by spores	Hyphae appeared on the surface of leaves, but no spores were produced
2	Minor symptoms with 6%–25% of the leaf covered by spores	Whitefly spot appeared, accounting for 25% of the disc area
3	Moderate symptoms with 26%–50% of the leaf covered by spores	Whitefly spot appeared, accounting for 75% of the disc area
4	Severe symptoms with 51%–75% of the leaf covered by spores	The disc area was covered by thick white spots, and abundant spores were produced
5	Severe symptoms with >76% of the leaf covered by spores forming a thick white layer	

Additionally, a *DI* was calculated using the formula:


$$ DI=\frac{\sum \left(s\times n\right)}{N\times S}\times 100 $$

Where:

- *s* is the number representing the disease grade,
- *n* is the number of leaves belonging to a specific disease grade,
- *N* is the total number of evaluated leaves,
- *S* is the number representing the maximum disease grade.

It is important to note that *DI* is an index of disease severity.

Based on *DI* values, the resistance evaluation standards are defined as follows: Immune (IM) for *DI* = 0; High Resistance (HR) for 0 < *DI* ≤ 25; Resistance (R) for 25 < *DI* ≤ 50; Susceptibility (S) for 50 < *DI* ≤ 75; and High Susceptibility (HS) for 75 < *DI* ≤ 100. IM, HR, and R are considered to be PM resistant, while S and HS are considered to be PM susceptible.

### Genetic inheritance analysis of PM resistance

To investigate the genetic inheritance of PM resistance, genetic populations were constructed using two parents ‘PI 164637’ and ‘HDZ’. Populations included P_1_ (‘PI 164637’), P_2_ (‘HDZ’), F_1_ (‘PI 164637’ × ‘HDZ’), F_2_ (F_1_ × F_1_), BCP_1_ (F_1_ × ‘PI 164637’), and BCP_2_ (F_1_ × ‘HDZ’). PM inoculation was performed at the two-true-leaves stage, and a resistance-to-susceptibility ratio was calculated based on the *DI* to determine the genetic inheritance of PM during the melon seedling stage. Furthermore, these plant populations were cultivated in a greenhouse at Sanya South Breeding Research Institute from November 2021 to February 2022. Given relatively high temperatures and humidities, PM occurs here annually. Plants were managed following standard horticulture procedures, with no PM prevention or control applied after pollination. Subsequently, PM incidence was investigated to explore the genetic inheritance of PM during the adult stage. Distinct phenotypes within the F2 population were used to establish the model of inheritance with the R package (SEA 2.0).

### DNA extraction and construction of bulked DNA pools

Leaves from P_1_, P_2_, F_1_, and F_2_ segregating populations were collected for DNA extraction, which followed a modified cetyl trimethyl ammonium bromide method [56]. DNA concentration was then determined using Nano Drop 2000 (Thermo Scientific, Waltham, MA, USA), and its quality was assessed through 1% agarose gel electrophoresis. Based on observed *DI* in the F_2_ segregating population, 25 plants showing extreme resistance and susceptibility were selected to construct two bulked DNA pools.

### BSA sequencing analysis and SNP identification

Sequencing was performed on the two bulked DNA pools and the parental DNA, which was outsourced to Novogene Bioinformatics Technology Co., Ltd. (Beijing, China). Raw reads were filtered to retain clean and high-quality reads. Filtered reads were then aligned to the melon reference genome (DHL92_4.0); identification of SNPs and InDels was performed using BCFtools (https://github.com/samtools/bcftools). The Δ(SNP-index) value was then calculated using the R package QTLseqr. A 99% confidence interval was set to determine the major QTLs associated with PM resistance in melon.

### QTL analysis and development and validation of InDel molecular markers

QTL analysis was performed to narrow down the candidate region associated with PM resistance based on BSA-seq results. Loci displaying insertions and deletions >5 bp were selected; discrepancies between parental sequences were verified using SAMtools [57]. Primers (~200 bp in length) that were flanked by SNPs or InDels were designed to amplify the specific locus using parental DNA as a template. PCR was performed with a 10-μl amplification system consisting of 1 μl of DNA template (50 ng·μl^−1^), 0.5 μl of each primer (10 μM), and 5 μl of 2 × Taq Master Mix (Vazyme, Nanjing, China), made up to 10 μl with ddH_2_O. The PCR reaction program involved 94°C for 3 min, followed by 35 cycles of 94°C for 30 s, 55°C for 30 s, 72°C for 45 s, a final amplification of 72°C for 10 min, and storage at 4°C. Resulting PCR products were subjected to 9% polyacrylamide gel electrophoresis [58] to visualize differences in band patterns between parents. Primers showing polymorphism between parents were selected to distinguish genotypes. Genetic mapping was constructed using IciMapping V3.3 software with a step size of 1 cM [59]. Inclusive Composite Interval Mapping (ICIM) was used to search for QTLs tightly linked to PM resistance, with an LOD value ≥5.0 regarded as available loci.

Polymorphic primers were validated using an F_2_ segregating population, and the accuracy of primer selection for resistant plant was then calculated. Selected primers also underwent further validation using a natural population of 30 inbred lines conserved by our laboratory.

### RNA extraction and RT-qPCR analysis

Parental plants ‘PI 164637’ and ‘HDZ’ were inoculated with *P. xanthii* at the seeding stage. Leaves were collected at 0, 24, 48, and 72 h after inoculation for total RNA extraction, which was performed using an RNA Isolation Kit (Cat #CW0581, Beijing, China) following manufacturer instructions. RNA integrity was assessed by 1% agarose gel electrophoresis, and purity was measured using the A260/A280 absorption ratio. Total RNA was then reverse-transcribed into cDNA using the PrimeScript RT reagent kit with genomic DNA Eraser (Takara, Dalian, China) following manufacturer instructions. Resulting cDNA served as template for RT-qPCR analysis. The RT-qPCR reaction system consisted of 1 μl of cDNA (1 μg·μl^−1^), 0.5 μl of each primer (10 μM), 5 μl of 2 × ChamQ Universal SYBR qPCR Master Mix (Vazyme, Nanjing, China), and 3 μl of ddH_2_O. Candidate genes were quantified by RT-qPCR with three biological and technical replicates. Their expressions were normalized using the *UBI-ep* gene; relative expression was calculated using the 2^−ΔΔCt^ method [60]. The expressions of candidate genes were normalized to their expression at 0 h after PM inoculation.

### Analysis of parental protein variants

Protein and CDS sequences were cloned from two parents and sequenced, and an alignment of the protein and CDS sequences was performed to detect variation using DNAMAN software. The protein sequences and amino acid changes were then input into SIFT tools (https://sift.bii.a-star.edu.sg/) to predict deleterious substitutions between parents according to the SIFT guidelines.

## Supplementary Material

Web_Material_uhae222

## Data Availability

The data supporting this article are available within the article itself and its supplementary material online.
