# Supplementary material for: Fine mapping and identification of candidate genes associated with powdery mildew resistance in melon (Cucumis melo L.)
Source: Hortic Res. 2024 Aug 12;11(10):uhae222. doi: 10.1093/hr/uhae222 (PMC11473854; doi:10.1093/hr/uhae222)
Supplement: Web_Material_uhae222 [file web_material_uhae222.zip › Supplemental Tables and Figures.pdf]

Table S1. Comparison of disease incidence (*DI)* or resistance evaluation values using three detection methods across 13 *C. melo* accessions.

Identification<br>*C. Melo* accession	Natural occurrence		Seedling inoculation		Detached leaf inoculation	
	DI	R/S	*DI*	R/S	*DI*	R/S
Iran H	77	S	79	S	100	S
Top Mark	78	S	76	S	100	S
Védrantais	75	S	58	S	100	S
PMR 45	0	R	2	R	0	R
PMR 5	0	R	0	R	0	R
WMR 29	0	R	0	R	0	R
Edisto 47	0	R	7	R	0	R
PI 414723	0	R	25	R	0	R
MR-1	0	R	2	R	0	R
PI 124111	0	R	33	R	0	R
PI 124112	0	R	5	R	0	R
PMR 6	0	R	0	R	0	R
Nantais Oblong	64	S	61	S	96	S

DI: disease index; R: resistance; S: susceptibility.

Table S2. Analysis of the mixed major gene plus polygenes model for PM resistance in melon.

Model	Log Max likelihood Value	AIC
0MG	87.2794	-170.5588
1MG-A	407.0524	-808.1048
1MG-NCD	250.7463	-493.4926
2MG-ADI	251.0398	-482.0796
2MG-CD	87.2784	-166.5568
2MG-EAD	87.2784	-168.5568

MG: major gene; A: additive; NCD: negative complete dominance; ADI: additive-dominant-epistasis; CD: complete dominance; EAD: equal dominance-additive-dominance; AIC: Akaike's information criterion.

Table S3. Suitability test for the mixed major-gene plus polygenes model for prediction PM resistance in melon.

Model	U_1^2	U_2^2	U_3^2	${}_nW^2$	D^n
1MG-A	22.10	1.79	165.39	12.99	0.43
	（0.00）*	（0.18）	（0.00）*	（0.03）*	（0.00）*

U_1^2，U_2^2 and U_3^2 : three uniformity tests, ${}_nW^2$: Smirnov test; D^n: Kolomogrov test. The P value in parentheses, * indicates significant difference at the 0.05 level.

Table S4. Summary of sequencing quality results.

Sample	Raw Reads (Mb)	Clean Reads (Mb)	Raw Base (G)	Clean Base (G)	Q20 (%)	Q30 (%)	GC Content (%)
‘PI 164637’	103.62	100.71	15.54	15.11	96.82	91.46	37.16
‘HDZ’	102.66	99.66	15.4	14.95	96.84	91.48	37.22
R	106.65	103.95	16	15.59	96.77	91.31	36.96
S	110.92	108.00	16.64	16.2	97.19	92.27	36.69

‘PI 164637’ is the female parent; ‘HDZ’ is the male parent; ‘R’ represents resistant DNA pool; ‘S’ represents susceptible DNA pool; Q20 represents the number of sequenced base that the accuracy more than 99% counts for total base; Q30 represents the number of sequenced base that the accuracy more than 99.9% counts for total base.

Table S5. Validation of chr06_indel_5047127 in a natural population.

Material number	Band type	*DI*	Resistance evaluation
CL_1	B	77.6	HS
CL_2	B	80.6	HS
CL_3	B	53.6	S
CL_4	B	64.4	S
CL_5	B	5.6	HR
CL_6	B	9.4	HR
CL_7	B	1.5	HR
CL_8	B	56	S
CL_9	B	58.8	S
CL_10	A	68	S
CL_11	B	6.4	HR
CL_12	A	78.6	HS
CL_13	B	10	HR
CL_14	B	54	S
CL_15	B	74.1	S
CL_16	B	33	R
CL_17	A	38.6	R
CL_18	A	15.6	HR
CL_19	A	10	HR
CL_20	B	54	S
CL_21	A	40.6	R
CL_22	A	68	S
CL_23	B	3.8	HR
CL_24	B	44	R
CL_25	B	18.2	HR
CL_26	B	10	HR
CL_27	A	31.3	R
CL_28	B	12.6	HR
CL_29	A	5	HR
CL_30	B	86.7	HS

A: PM-resistant type; B: PM-susceptible type.

Table S6. Functional annotation of candidate genes.

Gene ID	Start	End	Description	InterPro
MELO3C006693	5045134	5049511	Two-component response regulator	SANT/Myb domain
MELO3C006694	5050674	5053729	COBRA-like protein 7	COBRA, plant
MELO3C006695	5059327	5060441	leucine-rich repeat extensin-like protein 6	-
MELO3C006696	5068333	5070031	GATA transcription factor	Zinc finger, GATA-type
MELO3C006697	5075215	5079069	heparanase-like protein 1	Glycoside hydrolase, family 79
MELO3C006698	5080480	5081823	Protein of unknown function (DUF1218)	Modifying wall lignin-1/2
MELO3C006699	5084341	5084830	Unknown protein	-
MELO3C006700	5085848	5088743	Myb transcription factor	SANT/Myb domain
MELO3C028829	5093712	5094322	GATA zinc finger domain-containing protein 10-like isoform X2	-
MELO3C035461	5094061	5094327	GATA zinc finger domain-containing protein 10-like isoform X2	-
MELO3C006701	5099798	5105113	Pentatricopeptide repeat	Pentatricopeptide repeat
MELO3C006702	5108508	5111630	cAMP-regulated phosphoprotein 19-related protein	Endosulphine

Table S7. SIFT prediction analysis of candidate genes. Amino acids with $p < 0.05$ are predicted to be deleterious.

Gene	Substitution	SIFT score	SIFT prediction
MELO3C006693	T265A	0.18	Tolerated
	V334F	1	Tolerated
MELO3C006694	V262G	1	Tolerated
MELO3C006697	L285F	0.03	Deleterious
MELO3C006698	H258Q	0.5	Tolerated
	V262A	0.76	Tolerated
MELO3C006700	G197E	0	Deleterious
	W267K	0	Deleterious
MELO3C006701	E152G	0.06	Tolerated
	I375C	0.19	Tolerated
MELO3C006702	S11N	0.99	Tolerated
MELO3C028829	I16T	PSI-BLAST found no hits.	

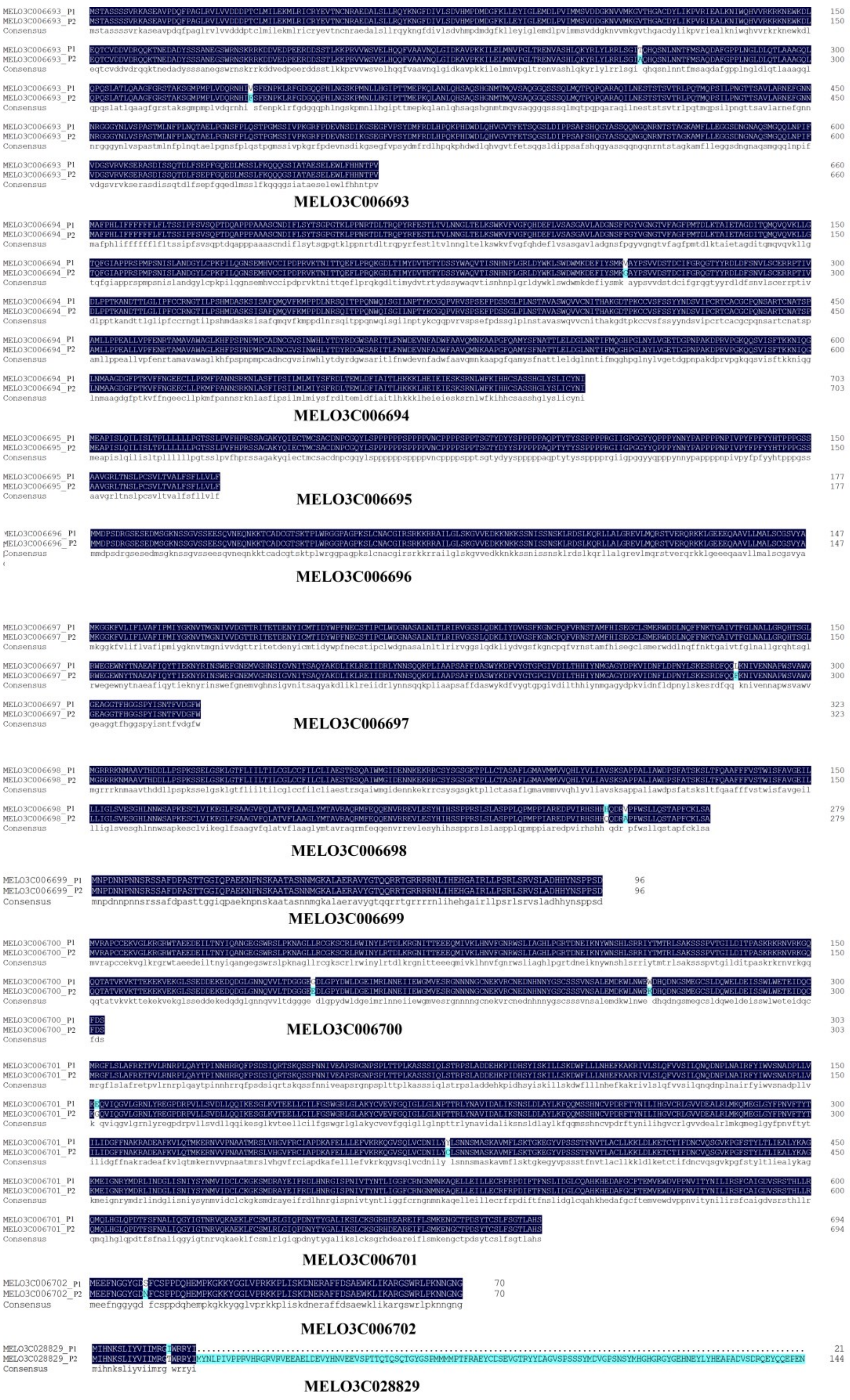

Figure S1. Alignment of candidate genes related to PM resistant between parental accessions ‘PI 164637’ and ‘HDZ’.

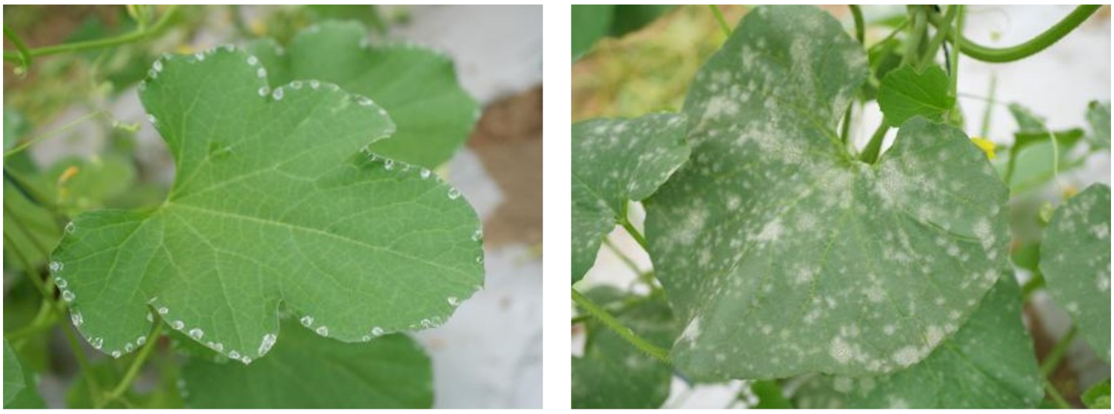

PI 164637 **HDZ**

Figure S2. Phenotypic responses of the two parents ‘PI 16437’ and ‘HDZ’ accessions to PM in the field.
